# Mechanisms of Anticancer Drug Resistance in Hepatoblastoma

**DOI:** 10.3390/cancers11030407

**Published:** 2019-03-22

**Authors:** Jose J. G. Marin, Candela Cives-Losada, Maitane Asensio, Elisa Lozano, Oscar Briz, Rocio I. R. Macias

**Affiliations:** 1 Experimental Hepatology and Drug Targeting (HEVEFARM), University of Salamanca, IBSAL, 37007 Salamanca, Spain; candelacives@usal.es (C.C.-L.); masensio002@usal.es (M.A.); elisa_biologia@usal.es (E.L.); obriz@usal.es (O.B.); 2 Center for the Study of Liver and Gastrointestinal Diseases (CIBERehd), Carlos III National Institute of Health, 28029 Madrid, Spain

**Keywords:** chemoresistance, chemotherapy, liver cancer, pediatric cancer

## Abstract

The most frequent liver tumor in children is hepatoblastoma (HB), which derives from embryonic parenchymal liver cells or hepatoblasts. Hepatocellular carcinoma (HCC), which rarely affects young people, causes one fourth of deaths due to cancer in adults. In contrast, HB usually has better prognosis, but this is still poor in 20% of cases. Although more responsive to chemotherapy than HCC, the failure of pharmacological treatment used before and/or after surgical resection is an important limitation in the management of patients with HB. To advance in the implementation of personalized medicine it is important to select the best combination among available anti-HB drugs, such as platinum derivatives, anthracyclines, etoposide, tyrosine-kinase inhibitors, *Vinca* alkaloids, 5-fluorouracil, monoclonal antibodies, irinotecan and nitrogen mustards. This requires predicting the sensitivity to these drugs of each tumor at each time because, it should be kept in mind, that cancer chemoresistance is a dynamic process of Darwinian nature. For this goal it is necessary to improve our understanding of the mechanisms of chemoresistance involved in the refractoriness of HB against the pharmacological challenge and how they evolve during treatment. In this review we have summarized the current knowledge on the multifactorial and complex factors responsible for the lack of response of HB to chemotherapy.

## 1. Introduction

Although hepatoblastoma (HB) is a rare tumor affecting less than 2 children per million (mainly under 3 years of age), this is the most common pediatric liver cancer [1]. Thanks to the enormous efforts of the scientific community, in part organized in international cooperative groups, important advances have been achieved over the last years regarding the understanding of HB biology and the accuracy of its diagnosis [2]. An example is the development of a system to assess the pretreatment extension of the tumor within the liver (PRETEXT). As a result, stratification of newly diagnosed patients has been proposed to help clinicians to select the most suitable treatment strategy for each patient. Surgical resection is potentially feasible in cases of standard-risk HB. Along with advances in surgical techniques, chemotherapy has contributed to achieve survival rates of up to 70–80% in contrast to the 20% obtained when patients were treated only with surgery [3]. It is of note that criteria for the management of patients with HB have been traditionally different among countries. Thus, in Europe, chemotherapy before surgery was recommended for all patients, whereas in USA, tumor resection followed by chemotherapy was allowed only if the tumor met strict resection criteria. The outcome after both therapeutic approaches seems to be similar. At present, the international collaboration of North American, European and Japanese pediatric oncology groups has laid to the Pediatric Hepatic International Tumor Trial (PHITT) with unified risk stratification, pathology definitions, surgical approaches, radiology definitions and treatment protocol [2]. The results may help to elucidate controversies, such as the intensity and duration of preoperative chemotherapy, the optimal surgical treatment for locally advanced tumors, the role of chemotherapy after liver transplantation and the treatment for the refractory or relapsed patients [4]. Most patients with HB respond to the standard treatment based on cisplatin and doxorubicin, however, the high-risk subgroup is characterized by marked chemoresistance and poor outcome [5].

According to the histology of the tumor, two subtypes have been distinguished: well-differentiated fetal HB and undifferentiated HB. Well-differentiated tumors have a completely epithelial phenotype, while poorly differentiated tumors are characterized by having a predominant mesenchymal component and a higher mitotic cell rate. The long-term survival of patients with undifferentiated HB is poorer than that of patients with well differentiated HB, especially in the case of small cell undifferentiated HB, an uncommon subgroup associated with an adverse outcome [6]. The Children’s Oncology Group advises surgery alone as the first-line curative therapeutic option for treating well-differentiated HB in early stage [7]. However, poorly differentiated tumors have worse prognosis and lower response to chemotherapy [8]. Even a small proportion of undifferentiated histology seems to confer a worse outcome [8]. The acquisition of mesenchymal features is associated with a more aggressive invasion, metastasis and chemoresistance [9]. Moreover, higher expression of some drug export pumps such as MRP4 has been found in more aggressive tumors and with a lower degree of differentiation [10].

The mechanisms responsible for the lack of response to chemotherapy in these patients are not well understood. As the nature of this phenotypic characteristic of tumors is multifactorial and complex, to facilitate its comprehension the mechanisms of chemoresistance (MOCs) have been classified in seven groups [11]: a reduction in the expression/function of plasma membrane solute carriers (SLCs) involved in drug uptake (MOC-1A) or an increased expression/function of export pumps belonging to the superfamily of ATP-binding cassette (ABC) proteins (MOC-1B), a reduced proportion of active *vs.* inactive drugs inside tumor cells due to changes in enzymes responsible for the activation of pro-drugs or the inactivation of drugs (MOC-2), changes in the expression/function of the molecular targets of anticancer agents (MOC-3), an increased ability of tumor cells to repair drug-induced DNA damage (MOC-4), decreased expression/function of pro-apoptotic factors (MOC-5A) or enhanced expression/function of anti-apoptotic proteins (MOC-5B), changes in tumor cell microenvironment that reduce the effectiveness of drugs (MOC-6) and activation of epithelial-mesenchymal transition (EMT) (MOC-7). Together with interindividual variations in the expression levels of genes involved in MOCs, the presence of genetic variants can also affect the response to chemotherapy [12]. In the present review we have revised the available information regarding the role of these MOCs in the lack of response to drugs commonly used in first- and second-line regimens of HB treatment, i.e., platinum derivatives, anthracyclines, etoposide, tyrosine-kinase inhibitors (TKIs), *Vinca* alkaloids, 5-fluorouracil (5-FU), irinotecan, bevacizumab and nitrogen mustards. 

## 2. Mechanisms of HB Resistance to Platinum Derivatives

**Figure 1:**
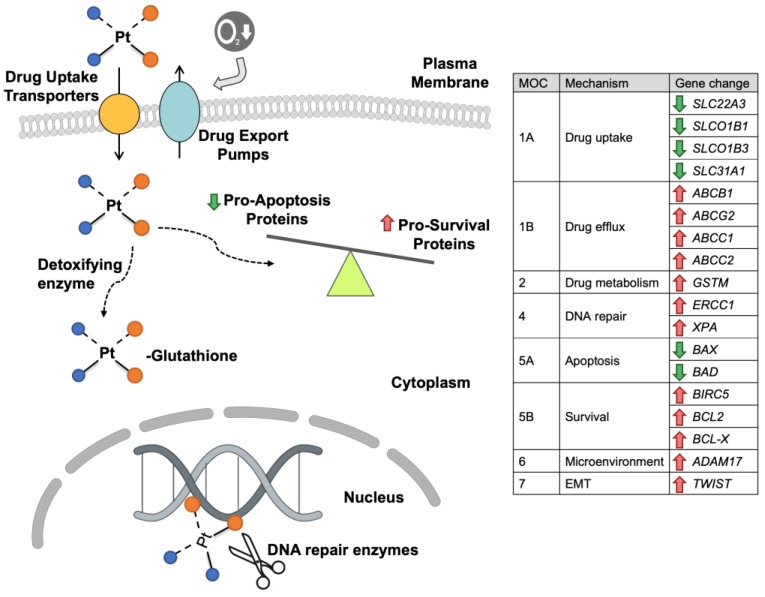
Schematic representation of mechanisms involved in chemoresistance (MOC) of hepatoblastoma to platinum derivatives. The table indicates the change in hepatoblastoma of the expression levels of genes involved in the chemoresistance to platinum derivatives: uptake transporters (MOC-1A), export pumps (MOC-1B), detoxifying enzymes (MOC-2), DNA repair enzymes (MOC-4), pro-apoptotic proteins (MOC-5A), anti-apoptotic proteins (MOC-5B), tumor cell microenvironment (MOC-6) and epithelial-mesenchymal transition (EMT) (MOC-7).

Platinum-based anticancer drugs are commonly administered in combination regimens used for the treatment of several solid tumors, including liver cancers [13]. Cisplatin was the first described platinum derivative and is a key drug in HB treatment, leading in some cases to improved resectability and longer patient survival. Other platinum derivatives, such as carboplatin and oxaliplatin, have been also used in clinic because both induce lower toxicity, but they are also less active than cisplatin. These drugs induce cell death mainly by irreversible DNA damage. However, it has been demonstrated that their cytostatic effects are also due to interactions with plasma membrane and cytoplasmic components [14]. In aqueous solution, platinum derivatives are hydrolyzed, which results in the generation of several molecular species with different charge and reactivity. There is limited information regarding the mechanisms involved in HB resistance to platinum derivatives (Figure 1). 

In the case of MOC-1, members of the SLC22A family of organic cation transporters (OCTs) have been involved in the uptake of platinum derivatives [15], but only OCT1 and OCT3 (*SLC22A1* and *SLC22A3* genes, respectively) are expressed in liver cells. Since OCT3 expression levels correlated with cisplatin intracellular accumulation and sensitivity in HB-derived HepG2 cells, OCT3 has been suggested to play a role in cisplatin resistance [16]. The formation of different metabolites justifies that the transport of these drugs involves several transport systems. In vitro and in vivo studies have shown that organic anion transporting polypeptide 1B1 (OATP1B1) and OATP1B3 (*SLCO1B1* and *SLCO1B3* genes, respectively) also mediate the cellular uptake of cisplatin, carboplatin and oxaliplatin [17]. However, there is some controversy because previous studies have failed to demonstrate the ability of OATPs to take up these platinum derivatives [18,19]. In a recent study we have reported that the levels of *SLCO1B1* and *SLCO1B3* mRNA are lower in HB tissue than in the adjacent non-tumor liver tissue. In addition, lower expression levels of both genes in these tumors correlated with worse prognosis [10]. This suggests that OATP1B1 and OATP1B3 may play a role in the uptake of platinum derivatives by HB and hence in the response to these drugs. The copper transporter CTR1 (gene *SLC31A1*) is also involved in the uptake of cisplatin, carboplatin and oxaliplatin. The loss of CTR1 results in the development of cisplatin resistance [20,21]. Although a time-dependent downregulation of CTR1 has been observed in human ovarian cells exposed to cisplatin [22], this behavior has not been seen in HB derived cells [10].

The overexpression of ABC transporters that mediate the efflux of anticancer agents is one of the main mechanisms accounting for multidrug resistance (MDR) [23]. The high expression of several ABC proteins, together with a marked chemoresistance, is a characteristic of embryonic stem cells from which HBs are originated [24]. Although it is believed that MDR1 is not a cisplatin transporter, two independent studies that analyzed by immunohistochemistry MDR1 (*ABCB1*) expression in resected primary and recurrent HB tumor specimens revealed an upregulation of this export pump after standard chemotherapy [25,26]. Moreover, upregulation of *ABCB1* was observed after treatment of mice bearing HB xenografts with cisplatin [27]. However, another study comparing protein expression in needle biopsies obtained before chemotherapy and in the surgically resected tumors have reported no changes in MDR1, but an increased expression of the breast cancer resistant protein, BCRP (*ABCG2*) [28]. In any event, the expression of *ABCB1* and *ABCG2* in HB specimens is higher than that found in healthy liver [29]. MDR-associated protein 2 (MRP2, *ABCC2*) is highly expressed in the apical membrane of hepatocytes and can reduce intracellular concentrations of cisplatin [30]. Increased MRP2 protein levels have been found in cisplatin-resistant HepG2 cells compared to wild-type cells [31]. MRP2 is highly expressed in HB biopsies [29] and in hepatocellular carcinoma (HCC), where MRP2 expression has been inversely associated to the efficacy of cisplatin-based chemotherapy [32]. Regarding other MRPs, upregulation of MRP1 (*ABCC1*) has been found in 50% of recurrent HB tumors after chemotherapy compared with primary tumors [26]. High levels of *ABCC1, ABCC2, ABCC3* and *ABCC4* were found in HB cell lines and HB specimens, and interestingly, higher expression of *ABCC4*, but not of other *ABCC* genes was observed in HB subtypes with a more aggressive phenotype (C2 vs. C1) [10]. However, as in the case of MDR1, both MRP1 and MRP4 do not transport cisplatin. Some information has suggested the possibility of inhibiting cisplatin export in hepatoma cells by reducing the activity of MRPs using glycyrrhizin and lamivudine [33]. Although it has been suggested that over-expression of ATPase Copper Transporting alpha (ATP7A) [34] and beta (ATP7B) [35] may confer resistance to platinum derivatives by enhancing their efflux, recent studies in HB-derived HepG2 cells have reported that ATP7B expression does not affect cisplatin sensitivity [16]. *ATP7A* mRNA levels were found enhanced in HB samples compared with healthy liver [29], although its relationship with survival after cisplatin treatment has not been investigated.

Concerning MOC-2, the group of enzymes glutathione S-transferases (GSTs) can play a role in cisplatin chemoresistance, since a higher conjugation of platinum derivatives with glutathione produces more inactive metabolites and a reduction of intracellular concentrations of active drugs [23]. Higher activity of GST alpha 1 (GST1A) has been associated with the resistance to cisplatin in several solid tumors [36], however, low expression of *GST1A* was found in HB specimens [29]. Upregulation of GSTmu (GSTM) was observed in two thirds of HB xenografts after treatment with cisplatin [37].

Owing to the mechanism of action of platinum derivatives, changes in proteins involved in DNA repair (MOC-4) can play an important role in chemoresistance. However, only one study has analyzed these genes in HB. Minor changes in the expression of genes included in MOC-4 were found in HB biopsies [29], but the same study demonstrated that the exposure of HepG2 cells to cisplatin upregulated excision repair cross-complementing 1 protein *(ERCC1)* and the product of *Xeroderma pigmentosum* group A (*XPA)* genes, suggesting that DNA repair mechanisms are induced by this pharmacological treatment.

Although the information concerning HB is scarce, changes in proteins involved in survival and apoptosis pathways (MOC-5) suggest that they may participate in HB resistance to chemotherapy. High levels of the anti-apoptotic proteins Bcl-2 and Bcl-XL, as well as low levels of the pro-apoptotic proteins Bax and Bad, were found in HuH6 and HepT1 cell lines [38]. Beside ABC-mediated sensitization, increased efficacy of cisplatin in vitro [39] and in a xenograft model of HB [40] has been reported by using the apoptosis sensitizer ABT-737, which inhibits both Bcl-2 and Bcl-XL. A consistent increase in the expression of survivin (*BIRC5* gene), a member of the inhibitor of apoptosis protein (IAP) family, has been found in liver tumors, such as HB, HCC and cholangiocarcinoma (CCA) [29]. The specific survivin inhibitor YM155 in combination with cisplatin markedly reduced proliferation in HepG2 and HuH6 cells and inhibited tumor growth in a xenograft mouse model in a more effective manner than monotherapy with cisplatin [41]. 

Regarding MOC-6, several studies have demonstrated tumor progression associated with high oxygen consumption and lower blood supply in tumor microenvironment, and that hypoxia enhances resistance to antitumor drugs. Thus, under hypoxia, HepG2 cells were more resistant to cisplatin than under normal conditions [42], which could be due, at least in part, to the up-regulation of the ABC pumps induced by hypoxia that would reduce intracellular cisplatin concentration [43]. ADAM17 is a transmembrane metalloproteinase found overexpressed in a variety of human tumors. Enhanced ADAM17 expression has been involved in cancer cell proliferation, chemoresistance and the development of a more malignant phenotype. In HepG2 cells ADAM17 expression was upregulated by hypoxia and associated with an increased resistance to cisplatin [44].

The loss of epithelial phenotype and the acquisition of mesenchymal features is associated with a more aggressive invasion, metastasis and chemoresistance (MOC-7). A recent study has reported that an intermediate-risk HB subtype, named C2B, has higher expression of the mesenchymal marker vimentin than the good-prognosis HB subtype C1 and the proliferative high-risk C2A tumors [9]. Several studies have demonstrated the relevance of EMT in resistance to platinum drugs. Both cisplatin- and carboplatin-resistant cancer cell lines have an upregulated mesenchymal gene expression signature compared with the parental cell lines [45]. The transcription factor twist family bHLH transcription factor 1 (TWIST) is known to induce EMT. An elevated TWIST protein expression found in liver tumors by immunohistochemistry correlated with MDR1 expression [46]. The same study described that doxorubicin-resistant HepG2 cells acquired an EMT phenotype and that knock-down of TWIST reversed EMT process and increased the sensitivity to drugs, including cisplatin [46]. Nanog, one of the transcription regulators required for maintaining the self-renewal capacity and undifferentiated state of embryonic stem cells, is considered an EMT marker and is highly expressed in tumors [47]. Disruption of Nanog expression in HepG2 cells resulted in reduced proliferation, altered migration and enhanced cisplatin effectiveness [48].

Clinical studies have revealed that the three risk-stratifying molecular subtypes of HB are characterized by differential activation of hepatic progenitor cell markers and metabolic pathways. One of these key regulatory elements is the nuclear factor (erythroid-derived 2)-like 2, also known as NFE2L2 or Nrf2, a transcription factor involved in the control of the expression of several genes accounting for cisplatin chemoresistance commented above, such as MRP2 [49], and is up-regulated in high-risk HB [50]. Interestingly, in relationship with MOC-6, under conditions of low glucose concentrations, an increase of Nrf2 expression in HepG2 cells has been observed [51].

## 3. Mechanisms of HB Resistance to Anthracyclines

In combination with platinum derivatives, the drugs most commonly used as first-line neoadjuvant and adjuvant treatments for HB are anthracyclines [52]. More precisely, doxorubicin, its epimer epirubicin and pirarubicin, a less cardiotoxic derivative, which has shown superior rates of survival among children in clinical studies [53]. Anthracyclines are amphipathic molecules with positive charge in aqueous solution that enter cancer cells via plasma membrane transporters rather than by simple diffusion [54].

**Figure 2:**
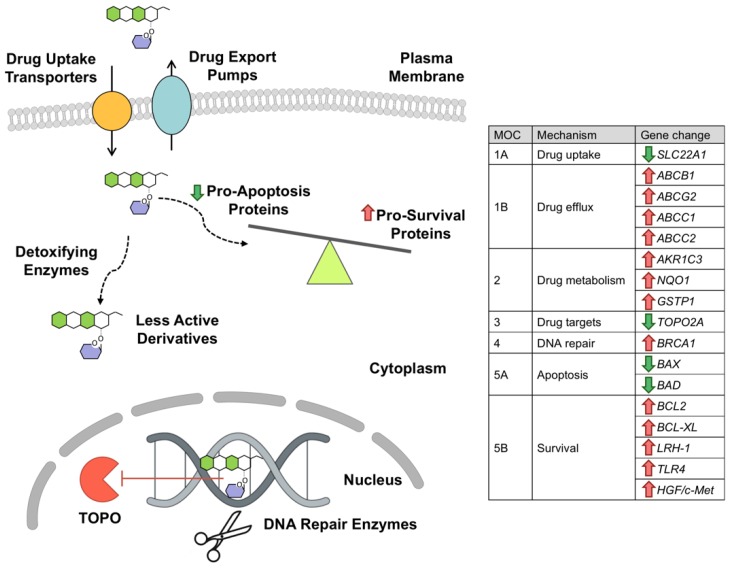
Schematic representation of mechanisms involved in chemoresistance (MOC) of hepatoblastoma to anthracyclines. The table indicates the change in hepatoblastoma of the expression levels of genes involved in the chemoresistance to anthracyclines: uptake transporters (MOC-1A), export pumps (MOC-1B), detoxifying enzymes (MOC-2), molecular targets (MOC-3), DNA repair enzymes (MOC-4), pro-apoptotic proteins (MOC-5A) and anti-apoptotic proteins (MOC-5B). TOPO, topoisomerase.

Several mechanisms have been involved in the resistance to anthracyclines (Figure 2). Regarding MOC-1, several OCTs participate in the cellular uptake of anthracyclines, such as OCT1 [55], OCTN1 [56] and OCT6 (*SLC22A16* gene) [57]. As happens in other primary liver tumors, a marked decrease in the expression of OCT1 in HB, when compared with the expression in adjacent non-tumor liver tissue, has been reported [10,29]. Decreased expression of OCT1 [58], OCTN1 [59] and OCT6 [57] has been associated with poorer sensitivity to doxorubicin and other drugs substrates of these carriers in different tumors. However, the importance of the downregulation of these transporters in the resistance of HB to anthracyclines has not been elucidated yet.

Overexpression of ABC export pumps plays an important role in the resistance to anthracyclines in several types of cancer, and an experimental model of MDR1 overexpression in HepG2 cells confirmed the relevance of this export pump in the resistance to doxorubicin [60]. The intrinsic expression of MDR1 in HB cells is high [26,27,29] and a clinically relevant observation in patients with HB is that MDR1 is usually upregulated during treatment with anthracyclines [26]. Increased MDR1 expression after treatment of mice bearing HB xenografts with doxorubicin has also been reported [27,37]. BCRP participates in the resistance of many tumors to anthracyclines [61]. An overexpression of BCRP has been found in tumors of patients with HB compared to control livers at mRNA [29] and protein [28] levels. Moreover, immunohistochemical analysis of needle biopsies obtained before chemotherapy and in the surgically resected tumors after chemotherapy based on doxorubicin and cisplatin found an increase in BCRP expression after pharmacological treatment in all analyzed patients, although it is not clear whether this was due to clonal selection or actual upregulation in individual tumor cells [28].

The expression of MRP1 is an important factor in resistance to anthracyclines in different types of cancer [62], however, the available data on the role of MRP1 in the clinical chemoresistance of HB are scarce. Overexpression of MRP1 in HCC was associated with a more aggressive phenotype with high similarity to that of hepatoblasts [63]. A high expression of MRP1 was reported in 1 out of 8 HBs analyzed [26]. Moreover, a relationship between upregulation of MRP1 and lower sensitivity to doxorubicin was found in HuH6 cells that were cultured in spheroids [64]. Since anthracyclines are substrates of MRP2 [65], MRP4 [66], MRP5 [67], MRP6 [68] and MRP7 [69], these pumps could potentially be also involved in the resistance to these drugs. However, regarding the expression of MRP2 in HB contradictory data have been reported; while MRP2 was found overexpressed in HB samples before chemotherapy as compared to normal liver [29], other studies have described similar levels of MRP2 in HB and in normal liver [28], with no clear change in MRP2 expression after chemotherapy with cisplatin and doxorubicin [28].

Enzymatic detoxification through several metabolic pathways contributes to resistance to anthracyclines (MOC-2). Carbonyl reducing enzymes, including aldo-keto reductases (AKR), carry out the transformation of aldehydic or ketonic groups into less potent hydroxyl metabolites. AKR1C3 is highly expressed in HepG2 cells [70]. NAD(P)H dehydrogenase quinone 1 is an enzyme encoded by the *NQO1* gene that participates in the metabolism of anthracyclines to generate derivatives with lower activity. Overexpression of *NQO1* has been found in the aggressive C2 subtype of HB, which could confer resistance to doxorubicin [71]. Moreover, enhanced GSTpi expression in HB cells was associated with lower sensitivity to doxorubicin [72].

Anthracyclines are DNA intercalating agents that can interact with their target TOPO2, stabilizing the enzyme-DNA complex and breaking the DNA strand, which eventually leads cancer cells to death by apoptosis activation. The decrease in the levels of TOPO2A found in HB [37] and the altered subcellular distribution [73] may be involved in the refractoriness of this cancer to anthracyclines (MOC-3). A significant decrease in the expression of TOPO2A occurring after the exposure of HB cells to doxorubicin could play a role in the loss of sensitivity to this drug [37].

The ability of anthracyclines to form formaldehyde-mediated DNA adducts contributes to their cytotoxic effect. NER and homologous recombination (HR) DNA repair pathways appear to be involved in the repair of anthracycline-DNA adducts (MOC-4) [74]. A relationship between upregulation of the breast cancer 1 (BRCA1) pathway, which regulates the double-strand DNA HR system, and resistance to doxorubicin in tumors from patients with HB classified in the subgroup C2A, with the worse prognosis and response to chemotherapy, has been found [9]. In addition, *ERCC1* downregulation in HepG2 cells with resistance to doxorubicin increased the sensitivity of these cells to the drug [75].

The p53 pathway is often impaired in cancer cells, increasing their resistance to the activation of apoptosis in response to chemotherapy (MOC-5). However, it has been found that doxorubicin-induced cell death involves pathways that are p53 independent [76]. Higher levels of the antiapoptotic proteins Bcl-2 and Bcl-XL and lower levels of the proapoptotic factors Bax and Bad were found in HuH6 cells compared to HepT1, being the former more resistant to doxorubicin than the latter [38]. In HB cells, the high activity of the signaling pathway involving the transcription factor GATA4 resulted in overexpression of Bcl-2 and resistance to doxorubicin-induced apoptosis [77]. Moreover, strategies to reduce the expression of Bcl-XL [78] or Bcl-2 [79] in HB cells increased the sensitivity to doxorubicin. High activity of the survival pathways of liver receptor homolog-1 (LRH-1) [80], Toll-like receptor-4 (TLR4) [81] and HGF/c-Met [82], which have been associated with resistance to doxorubicin, have also been found in HB.

Phenotypic changes during tumor progression associated to hypoxia (MOC-6) [83] and EMT (MOC-7) [84] have also been related to the sensitivity of HB cells to doxorubicin, mainly because these changes produce up-regulation of ABC transporters involved in the efflux of doxorubicin such as MRP1, MRP2 and BCRP [49].

## 4. Mechanisms of HB Resistance to Etoposide

Etoposide, also known as VP-16, is a lipophilic semisynthetic podophyllotoxin that in aqueous solution has neutral charge. This drug has been used in neoadjuvant regimens containing combinations of etoposide with cisplatin, anthracyclines and other drugs [85], after surgery [86] or in aggressive treatments against recurrent or advanced HB [87], obtaining, despite its high toxicity, promising results in terms of drug response and overall survival rates. 

Although etoposide is able to inhibit the transport function of several members of the OCT [88] and OATP [89] families, none of these carriers seem to be involved in etoposide uptake. Several ABC proteins, such as MDR1, MRP2 and MRP3 have been reported to be able to export etoposide [90], but the relevance of this mechanism in the resistance of HB to etoposide is not known. 

Etoposide is metabolized into less active catechol derivatives mainly through the cytochrome P450 (CYP) enzyme CYP3A4 that mediates etoposide demethylation occurring in liver cells [91]. Constitutive expression of CYP3A4 has been found in HepG2 cells [92]. Thus, active etoposide biotransformation by this enzyme may limit the sensitivity of HB to etoposide (MOC-2). The inactivation of etoposide and its metabolites by conjugation with glucuronide is mainly catalyzed by UDP-glucuronosyltransferases, which are highly expressed in HepG2 cells [93] and are presumably involved in HB chemoresistance to etoposide.

Etoposide interacts with TOPO2 and induces DNA strand breaks [94]. As described above, changes in TOPO2 levels and altered subcellular distribution decrease HB sensitivity to anthracyclines. This change in the molecular target of etoposide (MOC3) could also affect the response of HB to this drug. The same could happen with the mechanisms that repair the damage caused to DNA by the inhibition of TOPO2. DNA damage induced by this drug in cells can trigger apoptosis, which is inhibited in HB cells by the high expression of the antiapoptotic proteins Bcl-XL and Bcl-2 [38].

## 5. Mechanisms of HB Resistance to Tyrosine-Kinase Inhibitors (TKIs)

Sorafenib is a TKI able to inhibit several tyrosine kinase receptors (VEGFR, PDGFRβ, FLT-3 and Kit) and Raf kinases [95]. Currently, sorafenib is the first-line treatment of advanced HCC [96]. In HB xenograft models, sorafenib behaves as a potent inhibitor of tumor growth and angiogenesis [97]. In HB cell lines, its antiproliferative effect is enhanced by combination with cisplatin [98]. In pediatric HCC, sorafenib has shown promising results when administered in combination with cisplatin/doxorubicin [99]. At present, there are several ongoing clinical trials in patients with HB including sorafenib or regorafenib, a TKI analog of sorafenib (NCT03533582, NCT03017326, NCT02085148). 

In aqueous solution, sorafenib is in part in its cationic form, which implies that this drug needs plasma membrane transporters to reach its intracellular targets. OCT1 is mainly responsible for sorafenib uptake [58], but not of regorafenib [100]. Downregulation of OCT1 is a characteristic of liver tumors, such as HB [10,29], but also HCC [100] and CCA [101] (MOC-1). Expression levels of OCT1 and the localization in the plasma membrane of tumor cells have been associated with sorafenib response in patients with HCC [102]. 

Regarding export pumps, MDR1, MRP2 and BCRP have been found upregulated in HB tissue in comparison with the surrounding non-tumor tissue [29]. In vitro studies have demonstrated that ABC export pumps MDR1 [103] and BCRP [104] were able to reduce intracellular concentrations of sorafenib and were proposed as potential predictors for sorafenib sensitivity of HCC patients [105]. The presence of certain *ABCB1* and *ABCG2* polymorphisms were also associated with lower sorafenib plasma levels and better therapeutic response [106]. MRP2 has also been involved in resistance to sorafenib [107]. 

Sorafenib is mainly oxidized by CYP3A4, with the collaboration of CYP1A1 and CYP1B1 [108], whereas the contribution of CYP3A5 seems to be minor [109]. The important variability of CYP expression in tumors can affect the efficacy of TKIs (MOC-2). In vitro studies demonstrated that *CYP3A4* and *CYP1A1* levels were upregulated in HepG2 cells after cisplatin exposure [29]. Considering that sorafenib can be administered in combination with cisplatin/doxorubicin or as a second line treatment for patients with HB after the conventional treatment, this aspect deserves further investigation. 

Changes in molecular targets (MOC-3) of sorafenib have been involved in the resistance to this drug in different tumors [110,111]. Elevated expression of vascular endothelial growth factor receptor-2 (VEGFR-2) in tumor samples predicted poor outcome in advanced HCC patients treated with sorafenib [112]. Whether this biomarker could be useful in patients with HB has not been investigated, but overexpression of *VEGFR-2* has been observed in HepG2 cells after exposure to cisplatin [29]. 

The balance between pro- and anti-apoptotic factors can affect the response to pharmacological treatment with TKIs (MOC-5). Cellular inhibitor of apoptosis protein 1 (*cIAP1*), 3 (*BIRC3*) and survivin (*BIRC5*), which blocks caspase activation leading to apoptosis inhibition, have been found upregulated in HB biopsies [29]. In HepG2 cells sorafenib reduced upstream apoptotic parameters, but increased caspase-3 activation and DNA fragmentation [113]. Sorafenib-resistant HepG2 cells exhibited altered mRNA expression of anti-apoptotic proteins Bcl-2 and Mcl-1 affecting sorafenib/regorafenib response [114].

Because sorafenib exerts a strong inhibitory activity against EMT, chemoresistance due to phenotype transition (MOC-7) might play an important role in the response to this drug. HepG2 cells exhibit EMT and chemoresistance after long-term exposure to sorafenib [115]. HepG2 cells resistant to sorafenib showed increased metastatic and invasive ability compared to the parental cells, in part due to EMT, since the silencing of *Snail* blocked EMT and partially reversed MDR, invasion and metastasis [115]. A potent effect of sorafenib was observed in vitro in a cell line derived from a pediatric HCC and in HB cell lines, but only a moderate tumor growth inhibition occurred in vivo, which was associated with resistance during treatment [116]. This was accompanied by a decrease in E-cadherin and an enhanced activity of the transcription factor β-catenin, a major player in EMT.

## 6. Mechanisms of HB Resistance to Vinca Alkaloids

*Vinca* alkaloids are anti-mitotic and anti-microtubule agents used in the treatment of different tumors. Among these drugs, only vincristine and vinorelbine have been assayed in the treatment of patients with HB [117]. OATP transporters have been involved in the uptake of vincristine [118] and the expression of both *SLCO1B1* and *SLCO1B3* have been found lower in HB than in adjacent non-tumor tissue [10]. *SLC22A3* expression has been associated with the sensitivity to vincristine in kidney carcinoma cell lines [119], but no changes in the expression of this gene in HB have been found [10]. *Vinca* alkaloids are substrates of several ABC export pumps, such as MDR1 and MRPs [120,121]. In this sense, the reduction of *MDR1* [122] and *ABCC2* [123] expression by antisense RNAs increased the sensitivity of HepG2 cells to vincristine. *Vinca* alkaloids are metabolized by the liver mainly by CYP3A4 [124] and, as mentioned above, changes in CYP expression in tumors can affect the efficacy of these drugs. Although changes in the expression/function of β-tubulin isotypes have been also associated with acquired resistance to the *Vinca* alkaloids [121], there is no available information regarding this question in HB. 

## 7. Mechanisms of HB Resistance to 5-Fluorouracil (5-FU)

The pyrimidine base analog 5-FU acts as an antimetabolite due to the ability of its metabolites to compete with endogenous nucleosides for their incorporation into DNA/RNA and for inhibiting thymidylate synthase (TS, *TYMS*), a key enzyme in DNA synthesis. 5-FU is used in combination with conventional chemotherapy and other drugs in patients with HB [125]. The uptake of 5-FU by cancer cells is mainly carried out through the concentrative nucleoside transporter 1 (CNT1, *SLC28A1*) [126], whose expression is downregulated in HB [29]. Therefore, a deficient drug uptake can be involved in 5-FU chemoresistance. Regarding 5-FU efflux, several studies have found an association between the sensitivity of solid tumors to 5-FU and the expression of export pumps, such as MDR3 (*ABCB4*) [127], MRP5, and MRP8 (*ABCC11*) [128]. MRP5, which transports the monophosphorylated form of 5-FU [129], could contribute to the described chemoresistance, since MRP5 is overexpressed in HB cell lines [10]. The role of other transporters of 5-FU such as BCRP and MRP4 in the chemoresistance of HB to this drug remains to be elucidated. Dihydropyrimidine dehydrogenase (DPD) and thymidine phosphorylase (TP) participate in 5-FU metabolism [130]. DPD inactivates 5-FU, whereas TP plays a key role in the generation of 5-FU-monophosphorylated, the most active metabolite able to inhibit TS. DPD over-expression in several resistant solid tumors has been described, and TP has been proposed as a potential predictive marker of the response to 5-FU-based regimens [131], but the role of these enzymes in HB has not been investigated. TS levels have been associated with 5-FU sensitivity. An enhanced expression of *TYMS* has been described in HCC, but not in HB tumors [29] (MOC-2). Immunohistochemical analysis has revealed that p53 was abnormally accumulated in the cytoplasm of the HuH6 cells and HB samples [132]. This is interesting because p53 overexpression has been linked to the lack of response to 5-FU [133] (MOC-5). Hypoxia reduces 5-FU-induced inhibition of HepG2 cells proliferation by decreasing DNA damage [42] (MOC-6).

## 8. Mechanisms of HB Resistance to Nitrogen Mustards

Cyclophosphamide and isofosfamide are nitrogen mustards that have been used for the treatment of unresectable or recurrent HB, although with very poor response rates [134,135,136]. The anticancer activity of these drugs is based on its ability to alkylate and form DNA crosslinks, which prevents DNA strand separation and DNA replication. Both compounds are prodrugs that must be biotransformed in their active forms through hydroxylation by hepatic microsomal enzymes. MOCs affecting these drugs are poorly known. It has been demonstrated that isofosfamide, but not cyclophosphamide, is taken up by OCT2 [137], whose expression is restricted to kidney, but there is no information regarding the role of OCT1 and OCT3, that are expressed in liver cells, in the uptake of these drugs by HB cells (MOC-1A).

High expression of MRP1 in patients with breast cancer treated with cyclophosphamide has been related to shorter overall survival [138]. MRP1 may also be involved in MOC-1B for cyclophosphamide in HB, because this export pump is highly expressed in this type of tumor and is upregulated during the pharmacological treatment [26]. In HepG2 cells, MRP4 overexpression conferred resistance to isofosfamide [139], which was partially reversed by MRP4 inhibitors [140]. Interestingly, MRP4 expression was upregulated in tumors belonging to the most aggressive HB subtype C2 [10]. However, exposure of HepG2 cells to this drug had no effect on MRP4 expression [140]. 

Cyclophosphamide and isofosfamide are metabolized by enzymes of the CYP superfamily, mainly CYP2B6 and CYP3A4. Thus, a reduced activity of these enzymes is associated with chemoresistance. CYP enzymes are poorly expressed in end-stage liver diseases including HCC [141], but only CYP1A2 has been found downregulated in HB biopsies as compared to healthy liver [29] (MOC-2).

## 9. Mechanisms of HB Resistance to Bevacizumab

VEGF-A is an endothelial mitogen critical for the enhanced cell growth and angiogenesis that characterizes HB [142]. Bevacizumab is a recombinant monoclonal antibody that blocks angiogenesis by inhibiting VEGF-A. Bevacizumab, in combination with other drugs, has been proposed as a therapeutic option for the treatment of patients with recurrent HB who had no responded to more traditional agents [143,144]. The patients in whom it has been proven had a good response, despite the serious neurological and vascular adverse effects that the treatment can produce. The increase in serum levels of VEGF-A (MOC-3) [145] and the appearance of mutations in genes encoding proteins of the VEGF signaling pathway (MOC-5) [146] during bevacizumab treatment found in patients with colon cancer are potential predictive markers of a poor response to the drug in this type of cancer. Although in the case of patients with HB these mechanisms have not been studied, they could be shared with several types of cancer with poor response to this drug. An increased expression of VEGF, which can reduce the efficacy of bevacizumab, has been observed in HepG2 cells as a consequence of the hypoglycemia that characterizes the tumor microenvironment (MOC-6) [147]. It has been demonstrated that glucose deprivation increases VEGF expression in HepG2 cells through activation of the aryl hydrocarbon receptor (AhR)/activating transcription factor 4 (ATF4) pathway [51]. The endothelial PAS domain protein 1 (EPAS1) has also been identified as a transcription regulator of this pathway involved in over-expression of VEGF [51]. Higher expression of components of the extracellular matrix can sequester the active VEGF and has also been proposed as a MOC for therapies targeted against this growth factor [148].

## 10. Mechanisms of HB Resistance to Irinotecan

Irinotecan is a camptothecin that has been rarely used as chemotherapy in combination with vincristine in metastatic HB [149,150], in high-risk cases, despite its high toxicity. This drug has been described as a substrate of uptake transporters such as OCT1 [151], and ABC proteins such as MDR1 [152], MRP1 [153], MRP2 [65], MRP4 [139], MRP7 [69], and BCRP [61]. A reduction in drug content by down-regulation of OCT1 in HB [10] and upregulation of export pumps may lead to its chemoresistance in HB (MOC-1). In the liver, irinotecan is transformed into its active metabolite SN-38 by carboxylesterases (CES). A reduction in the expression of CES1, which has been found in patients with HB [29], could translate into lower levels of SN-38. In addition, UDP-glucuronosyltransferase 1A1 (UGT1A1), which is highly expressed in HepG2 cells [93], catalyzes the inactivation of SN-38, resulting in lower sensitivity to this drug (MOC-2). SN-38 inhibits TOPO1, causing irreversible DNA strand breaks. An increase in the expression levels of its molecular target has been reported in HB [29], which could be related to less response of HB to irinotecan (MOC-3).

## 11. Conclusions and Future Perspectives

The concept “resistome” is defined as the set of proteins, accounting for the lack of response to chemotherapy, that are expressed at each moment of tumor life. The present review describes how changes in the resistome, which includes a large variety of proteins involved in many aspects of tumor biology, could affect the efficacy of drugs that are commonly used to treat patients with HB. Nevertheless, as we are far from understanding all mechanisms involved in the poor response of HB to chemotherapy, some of them that are still being investigated have not been commented above. For instance, an intriguing question regards the role of Wnt/β-catenin signaling pathway in HB chemoresistance. Wnt/β-catenin pathway has been described as a key regulator of cell fate and proliferation during embryonic development that plays a main role in the generation, maintenance and differentiation of normal and malignant liver progenitor cells [154]. Aberrant deregulation of Wnt signaling has been described as a major mechanism of liver tumorigenesis [155]. In fact, up-regulation of Wnt signaling is a hallmark of HB, being this the malignancy with the highest frequency of β-catenin gene mutations in humans [155,156]. The alteration or activation of Wnt/β-catenin has been found to be associated with the increment in c-myc and cyclin D1 levels in HB tumors [157] that favors carcinogenesis. Moreover, Wnt signaling activation in HB samples has been associated with chemoresistance by an increase in the expression levels of MDR1 and matrix metalloproteinase-7 (MMP-7), when this signaling pathway is activated by telomerase. Additionally, the analysis of the clinicopathologic features of patients with HB revealed that those tumors with high expression of telomerase (that leads to a high Wnt signaling activity) showed poor response to neoadjuvant chemotherapy and low rates of tumor resectability [158]. 

Another limitation concerning our understanding of the problem of chemoresistance in HB comes from the fact that many of the observations have been done in cultured cells, mostly using the HB cell line HepG2, which recommends caution when extrapolating the *in vitro* findings to what is happening in the clinical setting of patients with HB. Moreover, it should be noted that an important part of the available information regarding the expression of MOC genes is derived from resected tumors with not accompanied data on the relationship with the response to pharmacological treatment. Thus, further studies in relapse and refractory tumor tissues must be performed to know exactly the gene expression patterns associated with HB chemoresistance. In addition, to determine the changes of the expression levels of the MOC genes, before and during pharmacological treatment, could help to predict the failure of chemotherapy. Based on this knowledge, it would be possible to develop novel medium- or high-throughput methods to define the chemoresistance signature of each tumor and consequently adapt the treatment aiming at reaching the maximal response in each case. Moreover, this information is crucial in the development of another line of research, which has already shown promising results. This is intended to sensitize the tumor to anticancer drugs by inhibition of one or several MOCs using pharmacological tools (e.g., chemosensitizers able to block ABC pumps), pharmacological approaches (e.g., nanoparticles encapsulating drugs) or gene therapy (e.g., targeted modulation of MOC gene expression). 

## Abbreviations

5-FU, 5-fluorouracil; ABC protein, ATP-binding cassette protein; AFP, α-fetoprotein; BCRP, breast cancer resistance protein; CCA, cholangiocarcinoma; DPD, dihydropyrimidine dehydrogenase; EMT, epithelial to mesenchymal transition; ERCC1, excision repair cross-complementing 1 protein; GST, glutathione S-transferase; HB, hepatoblastoma; HCC, hepatocellular carcinoma; HR, homologous recombination; MOC, mechanism of chemoresistance; MDR, multidrug resistance; NER, nucleotide-excision repair; OATP, organic anion transporting polypeptide; OCT, organic cation transporter; SLC, solute carrier; TOPO, topoisomerase; TP, thymidine phosphorylase; TS, thymidylate synthase; TWIST, twist family bHLH transcription factor 1; TKI, tyrosine-kinase inhibitor; VEGFR, vascular endothelial growth factor receptor.
